# Association between Non-Alcoholic Fatty Liver Disease and Chronic Kidney Disease: A Cross-Sectional Study

**DOI:** 10.3390/jcm9061635

**Published:** 2020-05-28

**Authors:** Takemi Akahane, Manabu Akahane, Tadashi Namisaki, Kosuke Kaji, Kei Moriya, Hideto Kawaratani, Hiroaki Takaya, Yasuhiko Sawada, Naotaka Shimozato, Yukihisa Fujinaga, Masanori Furukawa, Koh Kitagawa, Takahiro Ozutsumi, Yuki Tsuji, Daisuke Kaya, Akira Mitoro, Hitoshi Yoshiji

**Affiliations:** 1 Department of Gastroenterology, Nara Medical University, 840 Shijo-cho, Kashihara, Nara 634-8522, Japan; tadashin@naramed-u.ac.jp (T.N.); kajik@naramed-u.ac.jp (K.K.); moriyak@naramed-u.ac.jp (K.M.); kawara@naramed-u.ac.jp (H.K.); htky@naramed-u.ac.jp (H.T.); yasuhiko@naramed-u.ac.jp (Y.S.); shimozato@naramed-u.ac.jp (N.S.); fujinaga@naramed-u.ac.jp (Y.F.); furukawa@naramed-u.ac.jp (M.F.); Kitagawa@naramed-u.ac.jp (K.K.); ozutaka@naramed-u.ac.jp (T.O.); tsujih@naramed-u.ac.jp (Y.T.); kayad@naramed-u.ac.jp (D.K.); mitoroak@naramed-u.ac.jp (A.M.); yoshijih@naramed-u.ac.jp (H.Y.); 2 Department of Health and Welfare Services, National Institute of Public Health, 2-3-6 Minami, Wako, Saitama 351-0197, Japan; akahane.m.aa@niph.go.jp

**Keywords:** non-alcoholic fatty liver disease, chronic kidney disease, hepatic steatosis, obesity, hypertension, hyperuricemia, comorbidity

## Abstract

It is unclear whether the link between non-alcoholic fatty liver disease (NAFLD) and chronic kidney disease (CKD) is mediated by common risk factors. We aimed to elucidate the association between NAFLD and CKD using propensity score (PS)-matched analysis. We assessed 3725 Japanese individuals, excluding those with hepatitis B or C infection and men and women who consumed >30 and >20 g/day of alcohol, respectively. Of these, we enrolled 1097 Japanese subjects with NAFLD diagnosed by ultrasonography and 1097 PS-matched subjects without NAFLD. The prevalence of CKD was higher in subjects with NAFLD than in those without NAFLD before PS matching, but there was no significant difference between these groups in terms of CKD prevalence after PS matching. There was no difference in the prevalence of CKD between those with and without NAFLD in the subgroup analyses. Logistic regression analysis demonstrated that obesity, hypertension, and hyperuricemia were independent predictors of CKD, but NAFLD was not independently associated with CKD. In subjects with NAFLD, obesity, hypertension, and hyperuricemia were independent predictors of CKD. Thus, the link between NAFLD and CKD may be mediated by common risk factors. We recommend screening for CKD when patients with NAFLD have the aforementioned comorbidities.

## 1. Introduction

Chronic kidney disease (CKD) is a worldwide health problem that leads to a higher risk of dialysis, hospitalization, cardiovascular morbidity, and mortality [1,2,3]. The prevalence of CKD has been reported to be approximately 13% in the adult population [4,5]. There has been a recent dramatic increase in the prevalence of end-stage renal disease (ERSD), which is the final stage of CKD, because of the improvement in dialysis techniques and quality of medical care [6]. The number of chronic dialysis patients has been increasing over the last three decades, and the number of new dialysis patients has continuously increased in Japan [5]. Therefore, the latent CKD population appears to be enormous in Japan. Preventive plans mounted against CKDs will be essential to reduce the increase of patients with ESRD and consequent economic burdens. Recently, CKD has been considered as a risk factor for not only ERSD but also cardiovascular disease, even in the early stages of renal function [7]. Hence, it is very important to identify the risk factors of CKD to promote its prevention and early detection. In addition to CKD, non-alcoholic fatty liver disease (NAFLD) is one of the most common chronic liver diseases, and it represents a significant health burden worldwide. The prevalence of NAFLD has been estimated to be between 20% and 30% in the general population [8]. NAFLD includes a broad range of conditions, such as simple steatosis and non-alcoholic steatohepatitis, which could progress to cirrhosis and hepatocellular carcinoma. In addition, NAFLD is considered to be an independent risk factor for cardiovascular disease [9,10,11]. NAFLD has been strongly associated with obesity and has been considered a phenotype of metabolic syndrome (Mets) in the liver [12]. The components of Mets including obesity, diabetes, hypertension, and dyslipidemia are also important risk factors for the development and progression of CKD [1,13]. The possibility that CKD and NAFLD are linked has attracted attention in research interest since NAFLD is frequently accompanied by risk factors for CKD, such as insulin resistance, visceral obesity, type 2 diabetes, and Mets. Several studies have been reported that NAFLD increases the incidence of CKD [14,15,16,17,18]. In contrast, a recent cohort study found no relationship between NAFLD and CKD [19]. It is unclear whether the link between NAFLD and CKD is mediated by common risk factors, or whether NAFLD independently contributes to increasing the risk for CKD. Therefore, this study aimed to determine whether NAFLD itself is independently associated with CKD in a general population.

## 2. Materials and Methods

### 2.1. Study Design and Population

Our cross-sectional study used data from 4637 Japanese individuals (2461 men and 2176 women), who had consecutively undergone comprehensive annual examinations including ultrasonography for employee or resident health check-ups at Nara Health Promotion Center during 2012. Of these, 912 were excluded from the study because they were hepatitis B surface antigen- or anti-hepatitis C virus antibody-positive or consumed alcohol (men and women: >30 and >20 g/day of alcohol, respectively). The remaining 3725 subjects (1751 men, 1974 women; mean age: 53.4 ± 9.1 years, age range: 17–89 years) were included in this study.

This study was approved by the Ethics Committee of Nara Medical University (approval no. 1814-2). Informed consent was obtained in the form of opt-out from all participants prior to study.

### 2.2. Clinical and Laboratory Assessments

We used a standardized questionnaire to collect data including alcohol consumption, smoking history and status, and medical history. Smoking history and status were defined by using the Brinkman Index and answering the question whether you are a past or current smoker. The Brinkman Index measures the number of cigarettes smoked per day times smoking years. All subjects were examined following a 12-hour overnight fast. The body mass index (BMI) was calculated as weight (kg)/squared height (m^2^). Obesity was defined as a BMI ≥25 kg/m^2^ according to the criteria established by the Japan Society of Obesity. Resting blood pressure was measured using an automatic sphygmomanometer. Hypertension was defined as a systolic blood pressure (SBP) ≥140 mmHg, diastolic blood pressure (DBP) ≥90 mmHg, or current use of antihypertensive medications.

A high triglyceride level was defined as ≥150 mg/dL, and a high low-density lipoprotein (LDL) cholesterol level was defined as ≥140 mg/dL. Diabetes mellitus was defined as being present in patients on medication for diabetes mellitus and/or in those with a fasting blood glucose level ≥126 mg/dL. The glomerular filtration rate (GFR) was estimated from the Japanese Society of Nephrology CKD Practice Guide: estimated glomerular filtration rate (eGFR) (mL/min/1.73 m^2^) = 194 × (serum creatinine level (mg/dL)) − 1.094 × (age (y)) − 0.287. The product of this equation was multiplied by a correction factor of 0.739 for women. CKD was defined as an estimated GFR <60 mL/min/1.73 m^2^. Hyperuricemia was defined as ≥7 mg/dL.

Ultrasonography was performed with LOGIQ 7 using a 4 MHz convex array transducer (GE Healthcare, Waukesha, WI, USA). Fatty liver was defined by ultrasonography when there was hepatic parenchymal brightness and liver-to-kidney contrast. Since subjects were excluded from the study if they were hepatitis B surface antigen- or anti-hepatitis C virus antibody-positive, or consumed certain amounts of alcohol per day, those diagnosed as having fatty liver were considered to have NAFLD.

### 2.3. Statistical Analysis

We divided the subjects into two groups: subjects with NAFLD and those without NAFLD. There were potential confounding biases between the groups. In order to balance the bias, we calculated propensity scores (PSs) using the logistic regression model for the following covariates: sex; age; use of antihypertensive medications, antidiabetic medications, and lipid-lowering medications; history of cerebrovascular disease and heart disease; and smoking status (Brinkman Index). Subjects in both groups with similar PSs were matched in a 1:1 ratio using a caliper of 0.25 times the standard deviation of the PS. A standardized difference <0.2 was considered to indicate a negligible difference in the mean or prevalence of covariates between the groups.

Continuous variables were compared between the groups using the Student *t*-test, and categorical variables were compared using the chi-square test. Logistic regression analysis was used to assess the independent risk factors of CKD. The results are expressed as odds ratios with 95% confidence intervals. A *p*-value <0.05 was considered to be statistically significant. All calculations were performed using SPSS software (version 25, IBM Corp., Armonk, NY, USA).

## 3. Results

### 3.1. Clinical Characteristics

The baseline characteristics of the study population before and after PS matching are shown in Table 1. There were 2571 subjects without NAFLD and 1154 subjects with NAFLD before PS matching. The percentage of men; age; percentages of subjects using antihypertensive medications, antidiabetic medications, and lipid-lowering medications; percentage of those with a history of heart disease, and Brinkman Index were significantly higher in subjects with NAFLD than in those without NAFLD before PS matching. The PS technique matched 1097 subjects with NAFLD to 1097 subjects without NAFLD. After PS matching, those characteristics were not significantly different between subjects with and without NAFLD. All variables between subjects with and without NAFLD were within the threshold of acceptable balance (standardized difference <0.20).

### 3.2. Comparisons of Characteristics between Subjects with and without NAFLD before and after Propensity Score (PS) Matching

The comparison of characteristics between subjects with and without NAFLD before and after PS matching is shown in Table 2. In the groups both before and after PS matching, BMI, SBP, DBP, and the prevalence of hypertension and obesity were significantly higher in subjects with NAFLD than in those without NAFLD. Similarly, aspartate aminotransferase (AST), alanine aminotransferase (ALT), and gamma-glutamyl transferase levels were significantly higher in subjects with NAFLD than in those without NAFLD both before and after PS matching. Triglyceride, total cholesterol, and LDL cholesterol levels, and the prevalence of high triglyceride and high LDL cholesterol levels were significantly higher while the high-density lipoprotein cholesterol level was significantly lower in subjects with NAFLD than in those without NAFLD both before and after PS matching. The fasting glucose level and prevalence of diabetes mellitus were significantly higher in subjects with NAFLD than in those without NAFLD both before and after PS matching. Uric acid and the prevalence of hyperuricemia were significantly higher in subjects with NAFLD than in those without NAFLD both before and after PS matching. Before PS matching, the eGFR was significantly lower, and the serum creatinine level and CKD prevalence were significantly higher in subjects with NAFLD than in those without NAFLD. However, the serum creatinine level, eGFR, and prevalence of CKD were not significantly different between those with and without NAFLD after PS matching.

### 3.3. Comparisons of the Prevalence of Chronic Kidney Disease (CKD) in Subgroup Analyses after PS Matching

We stratified subjects by age, sex, and the risk factors of CKD, such as obesity, hypertension, high triglyceride level, high LDL cholesterol level, diabetes, and hyperuricemia, in subgroup analyses after PS matching (Table 3). Only eGFR showed significant differences with high triglyceride level (*p =* 0.038), normal LDL cholesterol level (*p =* 0.034), and high LDL cholesterol level (*p =* 0.025) between subjects with and without NAFLD. However, eGFR and CKD prevalence were not significantly different between subjects with and without NAFLD among those younger than 60 years of age; older than 60 years of age; male and female patients; or among those with obesity, hypertension, diabetes, and hyperuricemia. The prevalence of CKD was not significantly different between subjects with and without NAFLD among those with high triglyceride level and high LDL cholesterol level. In non-obese subjects, the eGFR and prevalence of CKD were not significantly different between subjects with and without NAFLD. Similar results were noted among subjects without hypertension, diabetes, and hyperuricemia and subjects with a normal triglyceride level. The prevalence of CKD was not significantly different between subjects with and without NAFLD among those with normal LDL cholesterol level.

### 3.4. Risk Factors of CKD in the Logistic Regression Analysis after PS Matching

Logistic regression analysis adjusted for age, sex, and the Brinkman Index demonstrated that obesity, hypertension, and hyperuricemia were independent risk factors of CKD (Table 4). However, NAFLD, high triglyceride level, high LDL cholesterol level, and diabetes were not independently associated with CKD.

### 3.5. Risk Factors of CKD in Subjects with and without Non-Alcoholic Fatty Liver Disease (NAFLD)

**Figure 1:**
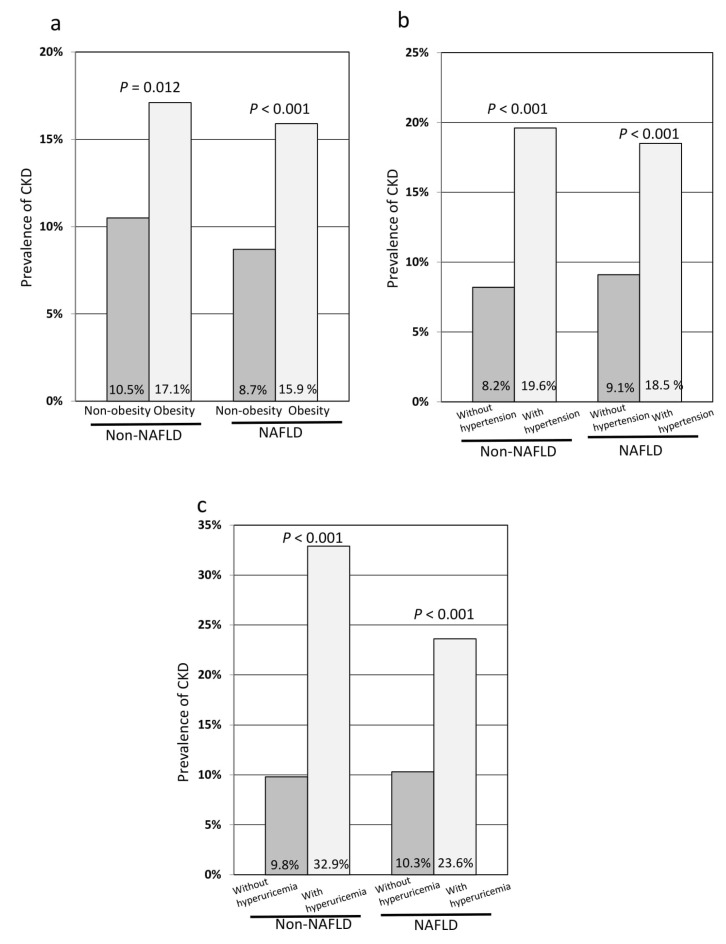
The prevalence of CKD in the subgroup analyses stratified by non-NAFLD and NAFLD. (**a**) Comparison of the prevalence of CKD between non-obese and obese subjects; (**b**) comparison of the prevalence of CKD between subjects with and without hypertension; (**c**) comparison of the prevalence of CKD between subjects with and without hyperuricemia. CKD, chronic kidney disease; NAFLD, non-alcoholic fatty liver disease.

In subjects with NAFLD, obesity, hypertension, and hyperuricemia were independent risk factors of CKD in the logistic regression analysis adjusted for age, sex, and the Brinkman Index (Table 5). However, high triglyceride level, high LDL cholesterol level, and diabetes were not independently associated with CKD. In subjects without NAFLD, hypertension and hyperuricemia were independent risk factors of CKD in the logistic regression analysis adjusted for age, sex, and the Brinkman Index (Table 6). However, obesity, high triglyceride level, high LDL cholesterol level, and diabetes were not independently associated with CKD. In subjects with NAFLD, the prevalence of CKD was significantly higher in obese subjects than in non-obese subjects (*p* < 0.001) (Figure 1a), in subjects with hypertension than in those without hypertension (*p* < 0.001) (Figure 1b), and in subjects with hyperuricemia than in those without hyperuricemia (*p* < 0.001) (Figure 1c). Similarly, in subjects without NAFLD, the prevalence of CKD was significantly higher in obese subjects than in non-obese subjects (*p* = 0.012) (Figure 1a), in subjects with hypertension than in those without hypertension (*p* < 0.001) (Figure 1b), and in subjects with hyperuricemia than in those without hyperuricemia (*p* < 0.001) (Figure 1c).

## 4. Discussion

This study showed that NAFLD itself is not an independent risk factor for CKD. The comorbidities of NAFLD such as obesity, hypertension, and hyperuricemia are independently associated with CKD. Logistic regression analysis adjusted for age, sex, and the Brinkman Index demonstrated that obesity, hypertension, and hyperuricemia were independent risk factors for CKD, but NAFLD was not independently associated with CKD. In subjects with NAFLD, obesity, hypertension, and hyperuricemia were independent risk factors for CKD. In addition, the prevalence of CKD was not significantly different between subjects with and without NAFLD in the subgroup analyses stratified by age, sex, and subgroups of subjects with and without the presence of comorbidities including obesity, hypertension, high triglyceride level, high LDL cholesterol level, diabetes, and hyperuricemia.

In this study, obesity and hypertension were independent risk factors for CKD in all subjects, and in subjects with NAFLD. In subjects with NAFLD, the prevalence of CKD was significantly higher in obese subjects than in non-obese subjects, and in subjects with hypertension than in those without hypertension. Obesity is the most common risk factor for NAFLD. Features of Mets are not only highly prevalent in patients with NAFLD, but components of Mets including obesity and hypertension also increase the risk of developing NAFLD [20]. Obesity is an independent risk factor for CKD, and it is associated with the development of proteinuria and pathologic findings of podocyte hypertrophy and focal segmental glomerular sclerosis even in the absence of hypertension. Hypertension has been defined as a risk factor for CKD. Systemic hypertension is transmitted to intraglomerular capillary pressure, which leads to glomerulosclerosis and loss of kidney function. Herein, hyperuricemia was also an independent risk factor for CKD in all subjects, and in subjects with NAFLD. Hyperuricemia leads to tubular injury, endothelial dysfunction, oxidative stress, and intrarenal inflammation. Additionally, when uric acid is present in the cytoplasm of cells or in the acidic/hydrophobic environment of atherosclerotic plaques, it is converted to a pro-oxidant, and it promotes oxidative stress, including cardiovascular disease through this mechanism, participating in the pathophysiology of human disease [21]. Recent studies reported that hyperuricemia is an independent predictor of CKD [22,23]. Uric acid concentration is an independent risk factor for kidney failure in earlier stages of CKD and has a J-shaped relationship with all-cause mortality in CKD [24]. A cross-sectional study reported that a higher serum uric acid level was associated with increased CKD prevalence in patients with hypertension [25]. Furthermore, in a longitudinal study it was demonstrated that a high serum uric acid level increases the risk of obesity. Hyperuricemia is also significantly associated with and independently predicts the risk for NAFLD. The serum uric acid level was significantly associated with the degree of steatosis and severity of lobular inflammation in NAFLD [26]. Hyperuricemia not only induces hepatic inflammation, but may also cause CKD in NAFLD. One of the most important causes of death in patients with NAFLD is the cardiovascular complications [27]. As mentioned, CKD is also considered an important risk factor for cardiovascular disease even in the early stages of renal function [7]. Therefore, NAFLD patients with hyperuricemia do not only have a high risk for CKD but also cardiovascular disease.

Diabetes is the leading cause of CKD. Among patients with type 2 diabetes mellitus who are initially free of proteinuria, the 20-year risk of diabetic nephropathy is 41% [28]. In the present study, there was no association between diabetes and CKD in all subjects or subjects with NAFLD. The numbers of subjects with diabetes were only 172 (7.8%) among all subjects and 113 (10.3%) among subjects with NAFLD. Therefore, the sample size was insufficient to elucidate the association between CKD and diabetes in all subjects and subjects with NAFLD by the methods used herein.

Since CKD and NAFLD have similar risk factors, it is necessary to demonstrate the independent association between NAFLD and CKD. A cross-sectional study in the United States showed a significant, positive association between NAFLD and prevalent CKD in univariate analysis. However, this association was attenuated after adjustment for features of Mets [29]. In another cross-sectional study in Taiwan, mild NAFLD was not significantly associated with CKD, but moderate to severe NAFLD was significantly associated with CKD after adjustment for age, sex, smoking, components of Mets, and the ALT level [30]. Since there are numerous risk factors for developing CKD including genetics, race, sex, age, smoking, obesity, history of cardiovascular disease, hyperlipidemia, and Mets, it is difficult to adjust for all the factors that affect the prevalence of CKD. In our study, before PS matching, the eGFR was significantly lower, and the serum creatinine level and the prevalence of CKD were significantly higher in subjects with NAFLD than in those without NAFLD. However, those values were not significantly different between subjects with and without NAFLD after PS matching.

Our study has several limitations. First, this study had a cross-sectional design that did not identify whether NAFLD itself develops and progresses to CKD. Second, we used only the eGFR for identification of CKD. However, in clinical practice and epidemiological studies, the eGFR is the most common variable evaluated for CKD, and the eGFR is used for the classification of CKD. Third, we did not assess the relationship between the severity of NAFLD and CKD. Liver biopsy is the gold standard for the assessment of hepatic fibrosis and inflammation severity. However, it has several limitations including sampling variability, invasiveness, complications and cost. Thus, it is recommended for patients who would benefit the most from diagnosis, therapeutic guidance, and prognostic information [20]. We could not perform liver biopsy because our sample size was large and the majority of the subjects were not eligible for liver biopsy. Qin et al. reported that liver stiffness assessed by transient elastography (TE) is a potential indicator of CKD in patients with NAFLD; such patients with a higher value of liver stiffness have an increased risk of CKD [31]. Therefore, the risk of CKD may increase with progression in the stage of liver fibrosis. TE is a clinically useful tool for identifying advanced fibrosis in patients with NAFLD. However, it cannot effectively diagnose bridging fibrosis or cirrhosis; therefore, to guide clinical decision-making, liver biopsy must be performed [32]. Recent studies reported that non-invasive fibrosis markers, the NAFLD fibrosis score (NFS) and fibrosis-4 (FIB-4) index are associated with the presence of CKD [33,34,35]. NFS is based on six variables including age, BMI, hyperglycemia, platelet count, albumin and AST/ALT ratio. FIB-4 index is based on age, platelet count, AST, and ALT. Because age is an independent risk factor for CKD, it is a cofounding factor in the analysis using the NFS and the FIB-4 index. These observations suggest that it may be difficult to accurately examine the relationship between the fibrosis stage of NAFLD and CKD in large studies. Fourth, NAFLD was diagnosed by ultrasonography. Although ultrasonography is a common method for diagnosing hepatic steatosis in clinical practice, it has limited accuracy for the detection of mild steatosis. We did not assess the relationship between the grade of steatosis and CKD. The controlled attenuation parameter (CAP) using TE provides a standardized non-invasive measure of hepatic steatosis [36] and is efficient to detect even low-grade steatosis [37]. It may be a useful tool to assess the relationship between the severity of steatosis and CKD. Fifth, we did not exclude autoimmune liver diseases such as autoimmune hepatitis and primary biliary cholangitis. However, due to their low prevalence, this may not significantly affect the results of this study. Lastly, most subjects were asymptomatic and able to engage in their work. Therefore, this study’s results may not be applicable to individuals who have liver cirrhosis or ERSD.

## 5. Conclusions

NAFLD itself was not an independent risk factor for CKD, but obesity, hypertension, and hyperuricemia, i.e., comorbidities of NAFLD, were independent risk factors for CKD. Thus, the link between these two diseases may be mediated by common risk factors. When patients with NAFLD have these comorbidities, the presence of CKD should be assessed. These results need further validation, including assessment for the causal relationship between the severity of NAFLD and CKD, and a large prospective study.

## Figures and Tables

**Table 1:** Baseline characteristics before and after propensity score (PS) matching.

	Before PS Matching				After PS Matching			
	Non-NAFLD	NAFLD	*p*-Value	STD	Non-NAFLD	NAFLD	*p*-Value	STD
*n*	2571	1154			1097	1097		
Sex, *n*, male	996 (38.7)	755 (65.4)	<0.001	0.51	714 (65.1)	702 (64.0)	0.592	0.02
Age, y	53.2 ± 9.5	54.1 ± 8.4	0.003	0.10	54.3 ± 9.2	54.0 ± 8.5	0.443	0.03
Use of antihypertensive medications	289 (11.2)	278 (24.1)	<0.001	0.45	214 (19.5)	225(20.5)	0.557	0.04
Use of antidiabetic medications	72 (2.8)	77 (6.7)	<0.001	0.45	49 (4.5)	58 (5.3)	0.372	0.09
Use of lipid-lowering medications	235 (9.1)	217 (18.8)	<0.001	0.41	155 (14.1)	169 (15.4)	0.400	0.05
Cerebrovascular disease	30 (1.2)	15 (1.3)	0.731	0.05	11 (1.0)	15 (1.4)	0.430	0.16
Heart disease	87 (3.4)	55 (4.8)	0.042	0.17	36 (3.3)	51 (4.6)	0.101	0.18
Smoking (BI)	113 ± 252	216 ± 342	<0.001	0.34	185 ± 320	205 ± 328	0.15	0.06

Values are expressed as the mean ± standard deviation or number (%). STD, standardized difference; BI, Brinkman Index; NAFLD, non-alcoholic fatty liver disease; PS, propensity score.

**Table 2:** Comparison of characteristics between subjects with and without NAFLD.

	Before PS Matching			After PS Matching		
	Non-NAFLD	NAFLD	*p*-Value	Non-NAFLD	NAFLD	*p*-Value
*n*	2571	1154		1097	1097	
BMI (kg/m^2^)	22.0 ± 2.7	25.8 ± 3.3	<0.001	22.4 ± 2.7	25.7 ± 3.2	<0.001
Obesity	320 (12.4)	635 (55.0)	<0.001	175 (16.0)	590 (53.8)	<0.001
Smoking			<0.001			0.008
Never	1846 (71.8)	626 (54.2)		678 (61.8)	613 (55.9)	
Past	476 (18.5)	352 (30.5)		261 (23.8)	321 (29.3)	
Current	249 (9.7)	176 (15.3)		158 (14.4)	163 (14.9)	
Systolic BP (mmHg)	121.9 ± 15.2	130.0 ± 14.5	<0.001	124.7 ± 14.7	129.5 ± 14.4	<0.001
Diastolic BP (mmHg)	72.7 ± 11.2	80.0 ± 10.8	<0.001	75.2 ± 11.0	79.2 ± 10.8	<0.001
Hypertension	520 (20.2)	459 (39.8)	<0.001	321 (29.3)	406 (37.0)	<0.001
AST level (IU/L)	21 ± 6	25 ± 10	<0.001	21 ± 5	25 ± 10	<0.001
ALT level (IU/L)	19 ±10	33 ± 21	<0.001	21 ± 9	33 ± 21	<0.001
GGT level (IU/L)	24 ± 22	41 ± 36	<0.001	28 ± 25	40 ± 35	<0.001
Triglyceride level (mg/dL)	87 ± 47	146 ± 92	<0.001	94 ± 44	146 ± 93	<0.001
High triglyceride level	192 (7.5)	399 (34.6)	<0.001	107 (9.8)	376 (34.3)	<0.001
Total cholesterol level (mg/dL)	210 ± 32	213 ± 34	0.004	208 ± 32	214 ± 93	<0.001
HDL cholesterol level (mg/dL)	65 ± 15	53 ± 11	<0.001	62 ± 15	53 ± 11	<0.001
LDL cholesterol level (mg/dL)	124 ± 29	135 ± 30	<0.001	125 ± 28	136 ± 30	<0.001
High LDL cholesterol level	726 (28.2)	478 (41.4)	<0.001	318 (29.0)	466 (42.5)	<0.001
Fasting glucose level (mg/dL)	95 ± 15	105 ± 19	<0.001	98 ± 16	105 ± 18	<0.001
Diabetes mellitus	90 (3.5)	134 (11.6)	<0.001	59 (5.4)	113 (10.3)	<0.001
Uric acid level (mg/dL)	4.8 ± 1.2	5.8 ± 1.2	<0.001	5.2 ± 1.2	5.8 ± 1.3	<0.001
Hyperuricemia	131 (5.1)	199 (17.2)	<0.001	85 (7.7)	191 (17.4)	<0.001
SCr level	0.74	0.81	<0.001	0.81	0.81	0.742
eGFR	74.7	73.1	<0.001	73.5	73.3	0.719
CKD	239 (9.3)	146 (12.7)	0.002	127 (11.6)	138 (12.6)	0.471

Values are expressed as the mean ± standard deviation or number (%). BMI, body mass index; BP, blood pressure; AST, aspartate aminotransferase; ALT, alanine aminotransferase; GGT, gamma-glutamyl transferase; HDL, high-density lipoprotein; LDL, low-density lipoprotein; SCr, serum creatinine; eGFR, estimated glomerular filtration rate; CKD, chronic kidney disease; NAFLD, non-alcoholic fatty liver disease; PS, propensity score.

**Table 3:** Comparison of the eGFR and prevalence of CKD in subjects with and without NAFLD in the subgroup analyses after propensity score matching.

	NAFLD Status	Number of Patients	eGFR	*p*-Value	CKD	*p*-Value
Age < 60 years	Non-NAFLD	816	74.9 ± 11.9	0.394	74 (9.1)	0.315
	NAFLD	825	74.3 ± 12.6		87 (10.5)	
Age ≥60 years	Non-NAFLD	281	69.3 ± 11.3	0.531	53 (18.9)	0.973
	NAFLD	272	69.9 ± 12.0		51 (18.8)	
Male sex	Non-NAFLD	714	72.6 ± 12.0	0.125	98 (13.7)	0.838
	NAFLD	702	71.7 ± 11.2		99 (14.1)	
Female sex	Non-NAFLD	383	75.1 ± 12.0	0.254	29 (7.6)	0.256
	NAFLD	395	76.2 ± 14.3		39 (9.9)	
Non-obesity	Non-NAFLD	922	73.9 ± 11.9	0.774	97 (10.5)	0.266
	NAFLD	507	73.7 ± 11.6		44 (8.7)	
Obesity	Non-NAFLD	175	71.2 ± 12.7	0.147	30 (17.1)	0.703
	NAFLD	590	72.8 ± 13.2		94 (15.9)	
Non-hypertension	Non-NAFLD	776	75.0 ± 11.6	0.655	64 (8.2)	0.554
	NAFLD	691	75.0 ± 12.2		63 (9.1)	
Hypertension	Non-NAFLD	321	69.9 ± 12.4	0.282	63 (19.6)	0.694
	NAFLD	406	70.9 ± 12.9		75 (18.5)	
Normal triglyceride level	Non-NAFLD	990	73.8 ± 12.1	0.373	110 (11.1)	0.541
	NAFLD	721	73.3 ± 12.3		87 (12.1)	
High triglyceride level	Non-NAFLD	107	70.7 ± 11.1	0.038	17 (15.9)	0.542
	NAFLD	376	73.4 ± 13.3		51 (13.6)	
Normal LDL cholesterol level	Non-NAFLD	779	73.9 ± 12.4	0.034	92 (11.8)	0.147
	NAFLD	631	72.5 ± 12.5		91 (14.4)	
High LDL cholesterol level	Non-NAFLD	318	72.5 ± 10.9	0.025	35 (11.0)	0.679
	NAFLD	466	74.4 ± 12.6		47 (10.1)	
Non-diabetes	Non-NAFLD	1308	73.4 ± 11.8	0.886	117 (11.3)	0.519
	NAFLD	984	73.4 ± 12.6		120 (12.2)	
Diabetes	Non-NAFLD	59	74.3 ± 15.0	0.473	10 (16.9)	0.863
	NAFLD	113	72.8 ± 13.1		18 (15.9)	
Non-hyperuricemia	Non-NAFLD	1012	74.1 ± 11.8	0.474	99 (9.8)	0.725
	NAFLD	906	74.5 ± 12.6		93 (10.3)	
Hyperuricemia	Non-NAFLD	85	65.9 ± 12.2	0.283	28 (32.9)	0.103
	NAFLD	191	67.5 ± 11.0		45 (23.6)	

Values are expressed as the mean ± standard deviation or number (%). eGFR, estimated glomerular filtration rate; CKD, chronic kidney disease; LDL, low-density lipoprotein; NAFLD, non-alcoholic fatty liver disease.

**Table 4:** Risk factors of chronic kidney disease.

	Odds Ratio (95% CI)	*p*-Value
Obesity	1.686 (1.246–2.281)	0.001
Hypertension	1.572 (1.183–2.088)	0.002
High triglyceride level	1.087 (0.780–1.516)	0.622
High LDL cholesterol level	0.833 (0.621–1.118)	0.223
Diabetes	0.922 (0.583–1.458)	0.728
Hyperuricemia	2.877 (2.029–4.080)	<0.001
NAFLD	0.832 (0.614–1.127)	0.220

Explanatory variables include age, sex, and the Brinkman Index. CI, confidence interval; LDL, low-density lipoprotein; NAFLD, non-alcoholic fatty liver disease.

**Table 5:** Risk factors of chronic kidney disease in NAFLD.

	Odds Ratio (95% CI)	*p*-Value
Obesity	2.104 (1.397–3.168)	<0.001
Hypertension	1.505 (1.021–2.219)	0.039
High triglyceride level	1.085 (0.728–1.616)	0.688
High LDL cholesterol level	0.717 (0.484–1.063)	0.098
Diabetes	0.950 (0.538–1.678)	0.860
Hyperuricemia	2.413 (1.537–3.788)	<0.001

Explanatory variables include age, sex, and the Brinkman Index. NAFLD, non-alcoholic fatty liver disease; CI, confidence interval; LDL, low-density lipoprotein.

**Table 6:** Risk factors of chronic kidney disease in non-NAFLD.

	Odds Ratio (95% CI)	*p*-Value
Obesity	1.229 (0.752–2.009)	0.411
Hypertension	1.662 (1.094–2.527)	0.017
High triglyceride level	1.227 (0.672–2.241)	0.505
High LDL cholesterol level	0.981 (0.631–1.524)	0.931
Diabetes	0.878 (0.406–1.898)	0.741
Hyperuricemia	3.884 (2.228–6.772)	<0.001

Explanatory variables include age, sex, and Brinkman Index. CI, confidence interval; LDL, low-density lipoprotein; NAFLD, non-alcoholic fatty liver disease.
